# Revealing the Mechanism of Aroma Production Driven by High Salt Stress in *Trichomonascus ciferrii* WLW

**DOI:** 10.3390/foods13111593

**Published:** 2024-05-21

**Authors:** Fangying Xian, Lin Yang, Huaqing Ye, Jinlin Xu, Xiaoping Yue, Xiaolan Wang

**Affiliations:** 1 School of Life Science (Health), Jiangxi Normal University, Nanchang 330022, China; vifyx@foxmail.com (F.X.); linyang2023@jxnu.edu.cn (L.Y.); 202240100759@jxnu.edu.cn (H.Y.); 202341500029@jxnu.edu.cn (J.X.); 2 College of Chemistry and Chemical Engineering, Jiangxi Normal University, Nanchang 330022, China

**Keywords:** douchi, methyl anthranilate, salt stress responses, transcriptome, food microbiology

## Abstract

Douchi is a Chinese traditional fermented food with a unique flavor. Methyl anthranilate (MA) plays an important role in formation of this flavor. However, the complicated relationship between the MA formation and the metabolic mechanism of the key functional microorganisms remains unclear. Here, we elucidated the response mechanism of aroma production driven by high salt stress in *Trichomonascus ciferrii* WLW (*T. ciferrii* WLW), which originates from the douchi fermentation process. The highest production of MA was obtained in a 10% NaCl environment. The enhanced expression of the key enzyme genes of the pentose phosphate pathway and shikimic acid pathway directed carbon flow toward aromatic amino acid synthesis and helped sustain an increased expression of *metK* to synthesize a large amount of the methyl donor *S*-adenosylmethionine, which promoted methyl anthranilate yield. This provides a theoretical basis for in-depth research on the applications of the flavor formation mechanisms of fermented foods.

## 1. Introduction

Douchi is a traditional Chinese fermented condiment that is widely consumed in China due to its unique flavor and texture [1]. A wide variety of volatile substances, such as esters, alcohols, and pyrazines, are involved in composing the flavor of douchi [2]; among them is methyl anthranilate (MA), which is an aromatic ester with a grapey, often sweet, warming aroma. It has several applications and is of economic value [3], with interest growing in the medical research field [4]. In addition, MA has been shown to bind to receptor proteins while inhibiting the development of inter-microbial signaling systems and reducing the formation of biofilms; thus, it has very promising applications in water treatment technology [3,5]. These applications demonstrate the important role of MA in the food industry and biomedicine.

Although MA plays an important role in many fields, it is mostly of plant origin [4]. However, due to the low content in plants and the complex and costly extraction process [4,6], there is no effective report on the commercial production of plant-derived MA, so the current commercial production of MA is dominated by chemical synthesis. Wang et al. [7] optimized the production of MA using a response surface methodology based on a chemical method. Unfortunately, the chemical method involves chemical principles such as Hoffman rearrangement, and has the disadvantages of a complex reaction mechanism, many by-products, and difficulty in product purification [7,8]. Therefore, it is not an environmentally friendly synthesis method. In contrast, microbial synthesis, as a more competitive and emerging approach, is a hot research topic nowadays, and this method has good sustainability, high productivity, low production cost, and high scalability. It can also effectively address the concerns about rapid global population growth and climate change [9,10]. Therefore, the use of microbial methods for MA synthesis will contribute to the green development of the industry, increase the sustainability of the synthesis process, reduce the cost of MA production, and improve production efficiency [4,11]. However, to the best of our knowledge, little research has been reported on MA synthesis using microorganisms. Luo et al. [4] constructed an MA biosynthetic pathway by introducing a maize-derived anthranilic acid, methyltransferase, to *Escherichia coli*. Kuivanen et al. [11] used metabolic engineering to modify *Saccharomyces cerevisiae*, which addressed the gap in the literature of MA synthesis by eukaryotes. In addition, a method for microbial-mediated (wood-rotting fungi, etc.) dimethyl anthranilate demethylation was published as a patent. This method can be used for demethylation to synthesize MA, but the need to add the exogenous substrate dimethyl anthranilate, which is toxic to most cells, and the requirement for the strict optimization and control of the reaction conditions have hampered the popularity of this method [12]. Interestingly, in the present study, we identified a yeast strain, *Trichomonascus ciferrii* WLW (*T. ciferrii* WLW), which produced high yields of MA in high-salt environments without the addition of exogenous substrates. Therefore, it is interesting to investigate the relationship between MA and environmental factors (salt stress). To the best of our knowledge, the regulatory mechanism of MA synthesis by microorganisms under high-salt conditions has not been elucidated. Therefore, unraveling the resistance mechanism of cells could provide theoretical support for improving the MA production capacity of cells, which could further promote industrial applications.

Thus, in this study, we aimed to reveal the mechanism of aroma production in *T. ciferrii* WLW driven by high salt stress. We examined the resilience of the aroma-producing yeast *T. ciferrii* WLW using a salinity tolerance test and detected the change in MA production capacity of this yeast with increasing salinity, and finally, we used transcriptome sequencing to further reveal the regulatory mechanism of the synthesis of MA by this yeast in response to salt stress. This study provides a theoretical basis for further understanding the potential application of *T. ciferrii* WLW yeast in the industry to provide effective strategies for subsequent metabolic regulation studies and the expansion of the production process.

## 2. Materials and Methods

### 2.1. Strain Source 

The strain *T. ciferrii* WLW used in this experiment was isolated from *Aspergillus*-type douchi (Nanchang Taoxiangyuan Seasoned Foods Co., Ltd., Nanchang, Jiangxi, China) in the previous work of our group. It was obtained from douchi samples by the dilution coating method, isolation purification, and ITS sequence identification. The strain was stored in a refrigerator at −80 °C in the laboratory at Guangdong Provincial Mycological Strain Conservation Center (GDMCC), with the conservation number GDMCC No.61319.

### 2.2. Salt Tolerance and Aroma Production Capacity in Salt Conditions Test

The strain was pre-cultured in yeast–peptone–dextrose (YPD) solid medium at 30 °C for 48 h. A sufficiently activated bacterial solution was serially diluted 10^5^-fold, and 100 mL each of solutions 10^3^–10^5^ was spread on YPD plates with salinities of 0% (control), 5%, 10%, 15%, and 20% (*w*/*v*, three biological replicates). The number of single colonies on the plates was counted (cfu/L) after 7 d. Subsequently, suitable single colonies were selected and cultured in the YPD liquid medium for 24 h, and this process was repeated once for proper activation. In order to study the trend of *T. ciferrii* WLW aroma production under salt stress and to determine the optimal salt concentration for aroma production, active seed inoculums were inoculated at 1% [13] (*v*/*v*) in YPD liquid medium (250/500 mL) at different salt concentrations (0%, 2%, 4%, 6%, 8%, 10%, 12%, 14%, and 16%, *w*/*v*) and assessed for MA production by high-performance liquid chromatography (HPLC).

### 2.3. Chemical Analysis

Supernatants were collected from centrifuged yeast liquid cultures and analyzed for MA titers using HPLC [4,14]. Prior to HPLC analysis, the samples were filtered through 0.22 μm syringe filters and the culture samples were diluted 1:1 with methanol (Aladdin Co., Ltd., Shanghai, China). The methanol used here to dissolve the samples contained 3 mmol L^−1^ of benzyl alcohol (Aladdin Co., Ltd., Shanghai, China) as an internal standard. The concentration of MA was determined using HPLC (Primaide, Hitachi, Co., Ltd., Tokyo, Japan) with a C18 column (5 μm, 4.6 × 150 mm, LaChrom Ultra, Hitachi, Co., Ltd., Tokyo, Japan) operated at 30 °C. The sample volume was 20 μL. The injection volume was 20 μL. The ratio of mobile phase A (aqueous solution containing 0.1% trifluoroacetic acid) to mobile phase B (methanol solution containing 0.1% trifluoroacetic acid) was 4:6, and the flow rate was set to 1 mL min^−1^. Detection was performed at 220 nm using a photodiode array detector in isocratic elution with a re-equilibration time of 2 min between each run.

### 2.4. Library Construction and Sequencing

The precipitate from the centrifuged yeast fermentation liquid was collected for the extraction of total RNA using TRIzol^®^ Reagent with three biological replicates, following the manufacturer’s instructions. RNA was purified using an RNA Purification Kit (Majorbio, Shanghai, China). Subsequent assessments of concentration and purity were performed using a Nanodrop2000 spectrophotometer. Agarose gel electrophoresis was utilized for integrity evaluation, and the RIN value was ascertained with an Agilent5300 Fragment Analyzer. Messenger RNA was isolated using oligo (dT) beads via poly(A) selection. Fragmentation was performed using a fragment buffer, followed by the synthesis of double-stranded cDNA using a SuperScript Double-Stranded cDNA Synthesis Kit (Invitrogen, Carlsbad, CA, USA) and random hexamer primers (Illumina, San Diego, CA, USA). End repair, phosphorylation, and A-tailing of the synthesized cDNA were performed according to the Illumina library construction protocol. The library size was selected for 300 bp cDNA target fragments on 2% low-range ultra-agarose. This was followed by 15 cycles of PCR amplification using Phusion DNA Polymerase (NEB). Sequencing was performed by Majorbio Bio-pharm Biotechnology Co., Ltd. (Shanghai, China) using a NovaSeq 6000 sequencer.

### 2.5. Transcriptome Data Analysis

Using default parameters, we trimmed and quality-controlled raw paired-end reads using fastp [15]. Next, we aligned the clean reads to the reference genome, which was obtained from our genome sequencing of *T. ciferrii* WLW, using HISAT2 software (http://ccb.jhu.edu/software/hisat2/index.shtml, v2.2.2.1, accessed on 14 December 2020) in the directional mode [16]. The mapped reads of each sample were assembled using StringTie3 with a reference-based method. To identify the differentially expressed genes (DEGs) between two different samples, we calculated the expression level of each transcript by the transcripts per million method. We used RSEM to quantify gene abundance [17]. We performed differential expression analysis using DESeq2 [18]. The DEGs with |Log_2_FC| ≥ 1 and Padj value < 0.05 (DESeq2) were considered as significant DEGs. In addition, we performed enrichment analysis based on the Gene Ontology (GO, https://geneontology.org/ accessed on 14 December 2020) and Kyoto Encyclopedia of Genes and Genomes (KEGG, https://www.kegg.jp/ accessed on 14 December 2020) databases to determine which DEGs were significantly enriched in GO terms and in terms of metabolic pathways compared to the transcriptome background at a Bonferroni-corrected *p*-value (Padj) < 0.05. Analyses of GO functional enrichment and KEGG pathways were conducted using GoATools and KOBAS [19], respectively.

### 2.6. Data Analysis

Experimental data are expressed as mean ± standard deviation. Data statistics were established using IBM SPSS Statistics 24 and Prism GraphPad 8.0.2. One-way analysis of variance (ANOVA) was employed to analyze the effect of change in salt concentration on survival, and generalized linear models were run to analyze the effects of independent variables (time and salt concentration) on MA production and the interaction effects of independent variables. Results were statistically significant when *p* < 0.05. 

## 3. Results and Discussion

### 3.1. Characterization of Salt Tolerance and MA Production in T. ciferrii WLW

**Figure 1:**
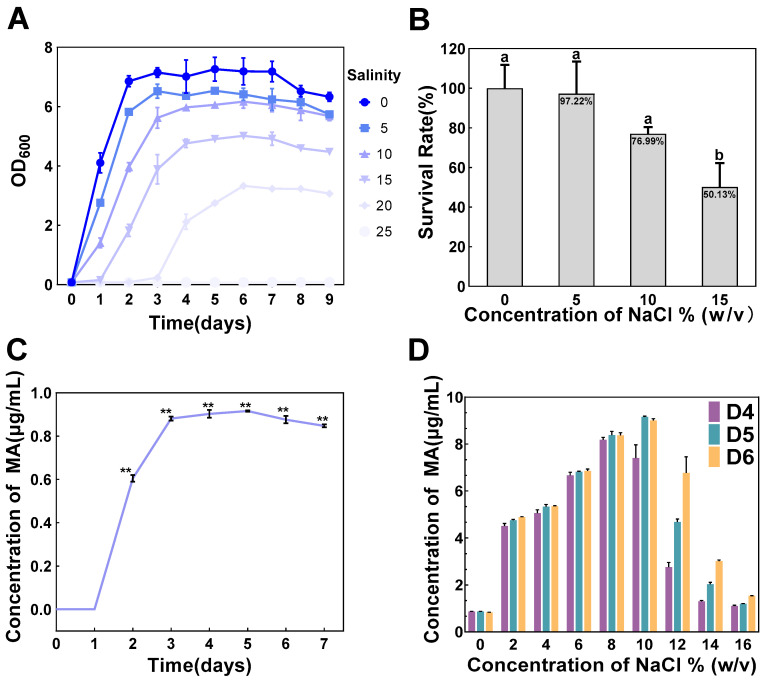
Effect of salt concentration on the ability of *T. ciferrii* WLW to survive and synthesize MA. (**A**) Curves of OD_600_ of *T. ciferrii* WLW in liquid medium at 0–25% (*w*/*v*) salinity; (**B**) changes in *T. ciferrii* WLW survival rate at 0–15% (*w*/*v*) salinity on solid medium plates (different letters above the columns indicate significant differences between groups, *p* < 0.05); (**C**) MA production curve of MA synthesized by *T. ciferrii* WLW in a salt-free state; (**D**) the change in aroma production of *T. ciferrii* WLW is in the range of 0%–16% (*w*/*v*) salinity. (D4–D6 represent Day 4–Day 6, respectively, and the value is mean standard deviation, *n* = 3. ** represents the level of significant difference at *p* < 0.01).

In general, microorganisms capable of growing in saline conditions above 15% (*w*/*v*) NaCl are defined as extremely halotolerant microorganisms [20]. The black yeast *Hortaea werneckii*, for example, can grow in NaCl concentrations ranging from 0% (*w*/*v*) to 32% saturation (*w*/*v*), and thus is classified as an extremely halotolerant fungus [21]. An aroma-producing strain, *Trichomonascus ciferrii* WLW (*T. ciferrii* WLW), previously screened by our group from high-salt fermented food environments, was tested for salt tolerance, and the results showed that *T. ciferrii* WLW had a fairly high survival rate and entered a growth plateau in 20% NaCl (*w*/*v*) aqueous saline environments on day 6 (Figure 1A), suggesting that it has the ability to grow stably. Using a salt-free condition as a control, the survival rate of solid medium plates in the 10% NaCl (*w*/*v*) range decreased, but not significantly (*p* > 0.05). In contrast, the change in the survival rate of the solid medium plates in the range of 15% (*w*/*v*) NaCl reached a significant level (*p* < 0.05), but the survival rate was still as high as 50.13% (Figure 1B). The above results indicated that *T. ciferrii* WLW has high salinity tolerance and is an extremely halotolerant microorganism. In addition, the high-salt conditions promoted an enhanced production of methyl anthranilate (MA) in *T. ciferrii* WLW, which led us to speculate that a correlation exists between the production of MA by *T. ciferrii* WLW and environmental factors (salt stress).

High-performance liquid chromatography (HPLC) is a technique capable of effectively detecting MA concentrations [4]. To further explore the optimal salt concentration for the synthesis of MA by *T. ciferrii* WLW, MA yield variations in a range of salt concentrations (0%–16% NaCl) were determined. The results showed that under salt-free conditions, the MA concentration reached a significant level on day 2 (*p* < 0.01) and entered a stabilization period on day 3 (Figure 1C), indicating that this strain has the ability to accumulate MA. A previous paper reported a method to generate MA using the natural demethylation of *Bacillus megaterium* [22]. However, this method requires the addition of exogenous substrates, which not only increases the cost but also the toxicity of the substrate to the microorganism. This means that the production process needs to be strictly controlled in terms of substrate concentration, thus limiting the production efficiency and discouraging the scale-up to commercial production. In contrast, *T. ciferrii* WLW-synthesized MA is clearly a more natural and less costly microbial pathway. In addition, MA production increased with increasing salinity (0–10% NaCl) compared to a salt-free environment (Figure 1D), and this effect of salt pressure driving the synthesis of MA reached a significant level (Table S1). The results showed that, on day 6, MA production at 10% (*w*/*v*) NaCl was approximately 9-fold higher than that at 0% salinity (Figure 1D). Interestingly, MA production decreased with increasing salinity when salinity exceeded 10% (Figure 1D). As a result, 10% (*w*/*v*) NaCl played the most important role in the optimal salt concentration for MA production by *T. ciferrii* WLW. In conclusion, salt pressure promoted MA synthesis in *T. ciferrii* WLW yeasts and MA production reached a maximum on day 6 at 10% salinity.

In fact, food microbial growth and metabolism are often influenced by the interaction of multiple external factors [23]. Generalized linear models are often used to reveal the interaction effects of independent variables on dependent variables [24,25]. Based on this model, in our study, there was a significant effect of salt concentration and time on MA yield effects (*p* < 0.001) (Table S1), and more importantly, there was still a significant effect on MA yield when time and salt concentration interacted (*p* < 0.001) (Table S1). Previous studies have demonstrated a significant effect of salt type and concentration on the resulting microbial community, as well as on the type and concentration of metabolites present in fermented foods [26,27]. This is similar to our findings, but the salt-tolerance mechanisms and molecular mechanisms of salt-pressure-driven MA synthesis in *T. ciferrii* WLW remain unclear and need to be further analysis.

### 3.2. Transcriptome Assembly and Difference Analysis 

**Figure 2:**
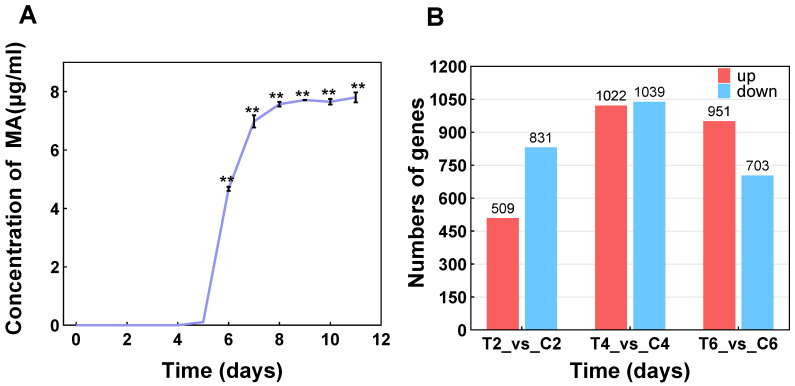
Trends in aroma production and genetic changes in *T. ciferrii* WLW under 10% salinity. (**A**) Aroma production curve of *T. ciferrii* WLW under 10% salinity; (**B**) number of significant DEGs for different days under salt stress. (** represents the level of significant difference at *p* < 0.01. “T2/C2”, “T4/D4”, and “T6/C6” represent data on days 2/4/6 for the treatment and control groups, respectively).

The transcriptome sequencing of *T. ciferrii* WLW at 0% and 10% (*w*/*v*) salinity was performed to determine the intrinsic relationship between MA production and salt pressure at the transcriptomic level. Based on the aroma production curve (250/500 mL) at 10% (*w*/*v*) salinity (Figure 2A), we selected samples from the 0% (*w*/*v*) NaCl control and 10% (*w*/*v*) NaCl-treated groups on Days 2, 4, and 6, and extracted RNA for the library construction and subsequent analysis. The reference genome was derived from the previously obtained whole-genome sequencing data of *T. ciferrii* WLW. Notably, after data quality control, the Q30 values of all sequencing data met the criteria (>90%) and the total mapping rates of the reference genomes were higher than 95% (Table S2). This result indicated that the quality of the sequencing data reached a qualified level and provided a reliable basis for subsequent analyses [28,29].

Subsequently, we further used the difference analysis mediated by DESeq2 [18] to define significant DEGs according to the rule of Padj value ≤ 0.05, |Log_2_FC| ≥ 1 [30]. As shown in Figure 2B, the comparison of the treatment group with the control group on Day 2 indicated a total of 1340 DEGs, of which 509 genes were upregulated and 831 genes were downregulated. On Day 4, a total of 2061 DEGs were obtained, of which 1022 genes were upregulated and 1039 genes were downregulated; on Day 6, a total of 1654 DEGs, of which 951 genes were upregulated and 703 genes were downregulated, were found. In summary, the number of differential genes under salt stress (10% NaCl) tended to fluctuate, suggesting that, compared to the controls, *T. ciferrii* WLW showed genetic differences at the transcriptome level under high-salt conditions, and the mechanism of salt-stress-driven MA synthesis may be a complex and dynamic process. Therefore, it is necessary to perform further differential gene analyses to reveal the molecular mechanisms by which salt stress drives *T. ciferrii* WLW to synthesize MA.

### 3.3. Mechanisms of T. ciferrii WLW Response to Salt Stress

In addition to the ability of *T. ciferrii* WLW to produce MA while tolerating high salt, the underlying molecular mechanism of the response to salt stress also attracted our attention. A series of studies related to the mechanism of salt tolerance have shown that membrane proteins, ion homeostasis, and transcription factors (TFs) are closely related to the strong salt tolerance of microorganisms under high-salt conditions [31,32,33]. Therefore, we used transcriptomic techniques to explore the reason for survival despite high-salt conditions from multiple perspectives, including the expression of key genes in terms of membrane proteins, ionic homeostasis, and salt-stress-related transcription factors.

**Figure 3:**
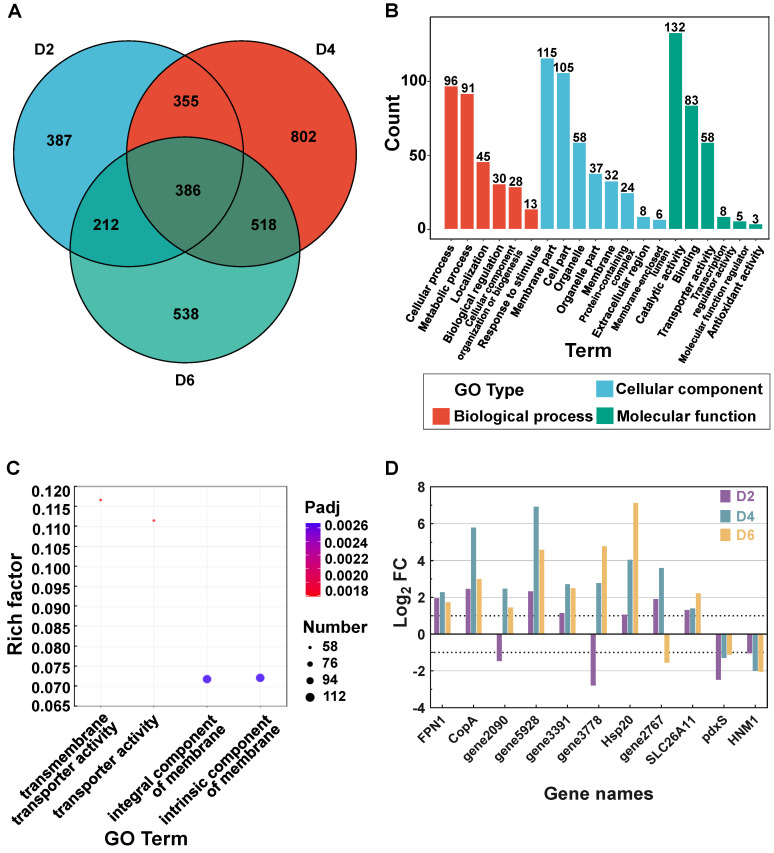
Days 2–6 of DEG counting and results of common DEG annotations and enrichment based on GO database. (**A**) Venn diagram of DEGs at Days 2–6. (**B**) Results of common significant DEGs at Days 2–6, annotated based on the GO database. (**C**) Bubble diagram of enrichment analysis of common DEGs based on GO database. Only terms with significant enrichment (Padj < 0.05) are shown. (**D**) Log_2_FC for 10 representative genes at Days 2–6.

Important genes related to the salt stress response in salt environments tend to show significant changes at the transcriptional level [34,35]. Therefore, we differentially analyzed the transcription of genes in conjunction with the Gene Ontology (GO, https://geneontology.org/ accessed on 14 December 2020) database. As shown in the Venn diagram (Figure 3A), more period-specific DEGs than common genes were present in *T. ciferrii* WLW at Days 2–6, suggesting that substantial metabolic changes may occur in *T. ciferrii* WLW under salt stress at different stages. Then, GO annotation and enrichment analysis were performed based on common genes at Days 2–6 to elucidate the persistent changes in *T. ciferrii* WLW transcript levels under high-salt conditions. The annotation results showed a high proportion of entries for cellular processes, metabolic processes, and localization activities (Figure 3B). These items are related to membrane transport, enzyme activity, ion localization, intracellular metabolism, and cell cycle changes, indicating that *T. ciferrii* WLW may alter its physiological and metabolic status by modulating genes related to transport, catalysis, bioregulation, localization, and organelle and cell membrane fractions to improve its tolerance to high-salt environments. In addition, the enrichment analysis of the common genes based on the GO database showed that four items related to membrane activity were significantly enriched (Padj < 0.05) (transmembrane transporter activity, transporter activity, integral component of membrane, and intrinsic component of membrane), suggesting that membrane-associated genes play an important role in the salt stress response (Figure 3C).

Because membrane proteins have an important effect on the environmental tolerance of cells [36], we investigated the transcription of relevant membrane protein genes using transcriptomic techniques. Notably, among the many DEGs identified in this study, some membrane protein genes continued to be significantly downregulated on Days 2–6 (Figure 3D). Amino acids are known to be important for resistance to salt stress [37]. One of these membrane protein genes is the pyridoxal phosphate synthase subunit gene *pdxS* (Figure 3D), which is a cofactor for amino acid catabolic enzymes in the pyridoxal phosphate metabolism and is involved in amino acid catabolic reactions, such as transamination and decarboxylation [38]. In the present study, *pdxS* was significantly downregulated at the transcriptional level at Days 2–6, which is similar to previously reported results [39]. In the present study, the persistent and significant downregulation of *pdxS* at the transcriptional level may be a result of the harsh high-salt environment, and to a certain extent, it may prevent amino acid degradation, thus helping to maintain the salt tolerance of *T. ciferrii* WLW. In addition, glycine betaine is involved in maintaining intracellular osmotic homeostasis and avoiding the toxicity of intracellular enzymes induced by high salt and inorganic ion concentrations [40]. The glycine betaine transmembrane transporter gene *HNM1* was significantly downregulated within Days 2–6, which may be due to the harsh, high-salt environment to which *T. ciferrii* WLW was subjected.

**Figure 4:**
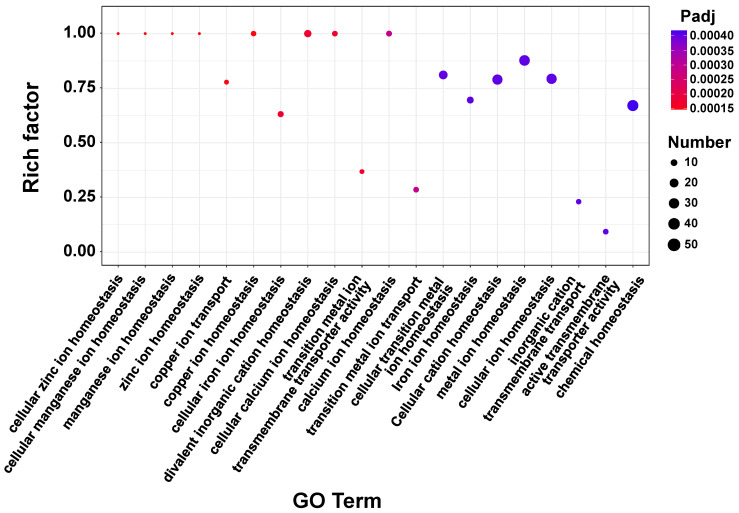
GO-enriched bubble plots of ion-homeostasis-related genes (only the first 20 significantly enriched terms are shown).

Inorganic ion homeostasis plays an important role in plant salt tolerance [41]. Thus, we performed further GO enrichment analysis of the genes involved in ion homeostasis. The top 20 significant entries were selected for further analysis, with the most significant ones being related to Zn, Mn, Cu, and Fe ion homeostasis (Figure 4). The genes for the ion homeostasis-related proteins *FPN1*, *CopA*, and *SLC26A11* were significantly upregulated in response to Fe, Cu, and Cl ions, respectively (Figure 3D). *SLC26A11* is usually reported to be an anion exchange channel with Cl channel effects [42,43]. The latter regulates the uptake and efflux of Cl and other anions from the cell membrane, thereby affecting cellular osmotic homeostasis, ionic balance, and pH regulation, which, in turn, enhances cellular salt tolerance [44]. Thus, the upregulation of Cl channels in *T. ciferrii* WLW may play a positive role in the salt stress response [45,46]. In addition, Ca ions are cofactors for a variety of metabolic enzymes, and calmodulin plays an important role in the salt stress response [47]. Therefore, as shown in Figure S1C, the regulation of Ca ion homeostasis and the overexpression of calmodulin-related genes contributed to salt tolerance in the yeast. To the best of our knowledge, the involvement of Cu/Fe/Mn ions in the response of fungi to salt stress has not yet been reported. Their significant enrichment in our study improves our understanding of the mechanisms by which yeast responds to abiotic stress. Calcium ions modulate the abundance of plasma membranes ZIP8 and ZIP14, thereby regulating the cellular uptake of Mn(II) [48], which acts as a coenzyme in metabolism. Iron and Cu ions have been reported to have strong redox activity and protein-binding capacity and participate in antioxidant processes, such as acting as antioxidant enzyme cofactors and inactivating free radicals [49,50]. Salt stress usually leads to oxidative stress [51]. In our study, Cu and Fe ion import-related genes (*gene5665* and *gene5666*) remained significantly upregulated (Figure S1A,B), suggesting that Fe and Cu ion homeostasis plays an important role in the response of *T. ciferrii* WLW to salt stress. In summary, the expression levels of ionic homeostasis-related genes were significantly altered under salt stress, indicating that ionic homeostasis plays an important role in the salt resistance of *T. ciferrii* WLW.

**Figure 5:**
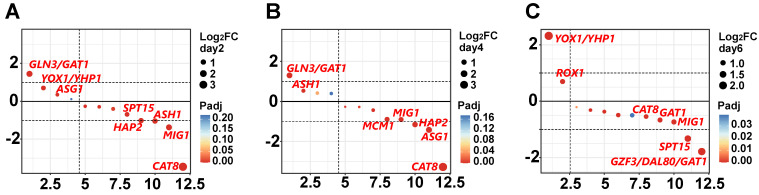
Bubble map of fold-change order of transcription factors. (**A**–**C**) correspond to the differential gene ordering of transcription factors on Days 2, 4, and 6, respectively. (Note: Horizontal coordinates indicate gene sequencing based on differential ploidy, and vertical coordinates indicate Log_2_FC).

In terms of TFs, which are important for regulating stress response [52], we analyzed the TF data obtained from DEGs under salt stress conditions based on the JASPAR database (http://jaspar.genereg.net/, accessed on 12 October 2019). Sixteen TFs were identified in our results, of which four families were known: C4-GATA-related factors, heterodimeric CCAAT-binding factors, other factors with up to three neighboring zinc fingers, and TBP-related factors (Table S3). The GATA transcription factors *Gln3* and *Gat1* regulate the transcription of permease and catabolite genes and inhibit nitrogen catabolism, playing an important role in the salt response of yeast [53,54]. Their significant upregulation during the pre-salt stress period (Days 2–4) (Figure 5A,B) might contribute to the high salt tolerance of *T. ciferrii* WLW. *YOX1/YHP1* are co-repressors of DNA transcription, and when DNA replication is stressed, high *YOX1* expression (Figure 5C) blocks cell cycle progression until it is able to overcome replication defects [55], thereby increasing cellular resilience to environmental stress [56], which might be an important reason for the high salt tolerance of *T. ciferrii* WLW. However, the downregulation of *CAT8* accelerates cell growth and glucose consumption, and alters the type of energy metabolism in yeast cells, in which many genes related to the mitochondrial respiratory chain are downregulated, leading to a decrease in aerobic respiration and the tricarboxylic acid cycle [57]. Our results were similar to this (Figure 5A,B), suggesting that salt stress might alter the energy metabolic composition of *T. ciferrii* WLW. Overall, TFs are strongly associated with salt resistance in *T. ciferrii* WLW.

In summary, *T. ciferrii* WLW regulates the expression of key genes in terms of membrane proteins, ionic homeostasis, and salt stress-related TFs to enhance salt stress capacity, thus laying the foundation for high MA production despite the high salt environment.

### 3.4. Timing Analysis of the MA-Synthesis-Pathway-Associated DEGs 

To reveal the mechanism of MA production driven by salt stress, a timing analysis of the MA-pathway-associated DEGs was performed based on the difference analysis. Several previous studies have shown that the MA de novo synthesis pathway flows through the glucose through glycolysis pathway (EMP), pentose phosphate pathway (PPP), shikimate pathway, and chorismate synthesis tryptophan pathway (trp biosynthesis) [4,11].

**Figure 6:**
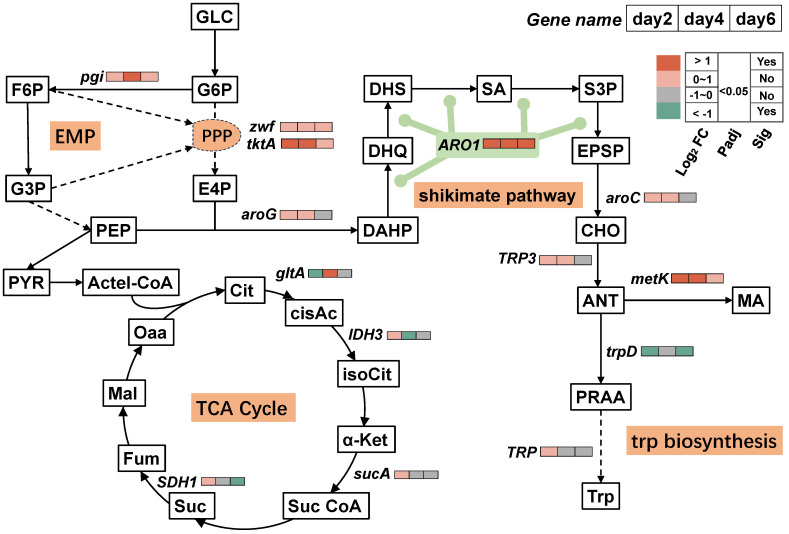
Expression of pathways related to MA synthesis. GLC: glucose; G6P: glucose-6-phosphate; F6P: fructose-6-phosphate; G3P: glyceraldehyde-3-phosphate; PEP: phosphoenolpyruvate; PYR: py-ruvate; TCA cycle: tricarboxylic acid cycle; E4P: Erythritol-4-phosphate; DAHP: 3deoxy-α-arabinoheptulosonate-7-phosphate; DHQ: 3-dehydroquinate; DHS: 3- dehydroshikimic acid; SA: shikimic acid; S3P: shikimate-3-phosphate; EPSP: 3-enol pyruvylshhikimate-5-phosohate; CHO: chorismite; ANT: anthranilate; MA: methyl anthranilate; PRAA: N-(5′-phosphoribosyl) anthranilate; Trp: tryptophan; Cit: citrate; cisAc: cis-Aconitate; isoCit: isocitrate; α-Ket: α-ketoglutarate; Suc CoA: succinyl CoA; Suc: succinate; Fum: fumarate; Mal: malate; Oaa: oxalo-acetate. (Note: Solid lines indicate one-step reactions, while dashed lines indicate multi-step reactions; the abbreviation ‘Sig’ is used to indicate the significance of genes).

As shown in Figure 6, in the case of glucose through EMP, normally, glucose is broken down by glycolysis and flows to the citric acid cycle (TCA cycle), the main pathway of energy synthesis, to synthesize energy with the aid of oxidative phosphorylation [58]. However, in the present study, three key regulatory enzyme genes of the TCA cycle, *gltA* (citrate synthase), *IDH3* (isocitrate dehydrogenase), and *SDH1* (succinate dehydrogenase), were significantly downregulated on Days 2, 4, and 6, respectively (Figure 6 and Figure S3 and Table S4). These results imply that the TCA cycle may be impaired and oxidative phosphorylation is reduced. Notably, the downregulation of the TCA cycle may help carbon flow toward the PPP and, thus, toward MA synthesis. 

Therefore, we found that the expression of the pathway genes in both the control and salt-treated groups and key gene of the PPP, *tktA*, which encodes ketolase, were significantly upregulated on Days 2 and 4 under salt conditions (Figure 6). The results suggest that salt pressure may be an important factor driving the overexpression of key genes.

The shikimate pathway, which plays an important role in MA synthesis, is the third pathway through which carbon flows to synthesize MA [4]. Notably, *ARO1*, a key enzyme gene in the shikimate pathway, encodes a pentafunctional AROM polypeptide that is responsible for positively catalyzing the five key steps of the shikimate pathway [59]. In our text, the expression of *ARO1*maintained a high differential fold value (Log_2_FC > 10) on Days 2–4 of salt stress (Figure 6 and Figure S2). This means that the high expression of the *ARO1* gene in *T. ciferrii* WLW in a saline environment is helpful for the forward progress of the shikimic acid pathway and may further promote carbon flow towards MA synthesis. The shikimate pathway is directly involved in the synthesis of aromatic amino acids (phenylalanine, tyrosine, and tryptophan) [60]. In addition to its important role in salt tolerance [61,62,63], more importantly, tryptophan synthesis is directly related to MA synthesis [4]. In our study, the upregulation of the shikimate pathway not only contributed to an improvement in cellular salt tolerance, but also aided in the direction of carbon flow towards MA synthesis. 

According to some previous reports, in the chorismate synthesis tryptophan pathway, the synthetic reaction of anthranilic acid (ANT) to MA is catalyzed by methyltransferase, and *S*-adenosylmethionine (SAM) is required as a methyl donor for the reaction [64,65]. Therefore, methyltransferases play an important role in MA synthesis. As a common coenzyme involved in methyl transfer reactions, SAM is used to synthesize MA from ANT by donating a methyl group to the ANT molecule, leading to MA formation [4]. In our study, we noticed that the upregulation of the shikimate pathway continued up to ANT and into the synthesis of MA, and the SAM synthase gene (*metK*) was significantly upregulated on Days 2 and 4 (Log_2_FC > 1, Padj < 0.05) (Figure 6), suggesting that *T. ciferrii* WLW has sufficient SAM at this stage, and that the methyltransferases on this metabolic branch from ANT to MA may be highly active, thus contributing to MA synthesis. This may be an important reason why *T. ciferrii* WLW produces a high amount of MA under salt stress. Noteworthily, the related genes in tryptophan biosynthesis located after ANT, *trpD* (anthranilate phosphoribosyl transferase) and *TRP* (tryptophan synthase), were consistently downregulated (Figure 6), suggesting that *T. ciferrii* WLW chooses to downregulate tryptophan synthesis under salt stress. As a result, this could help in the synthesis and accumulation of MA. In addition, in the tryptophan catabolism–kynurenine pathway, the gene encoding the enzyme for catalyzing the conversion of formyl anthranilate to ANT was consistently upregulated on Days 2 and 4, which may also contribute to the accumulation of ANT and further aid in the synthesis of MA (Figure 6). 

In summary, the above-mentioned genes in the MA synthesis pathway play a key role in increasing MA production and can be activated under salt pressure. *T. ciferrii* WLW regulated the expression of key genes (e.g., *metK*, *tkt*, and *ARO1*) in the TCA cycle, the PPP, the shikimate pathway, and tryptophan biosynthesis in response to salt stress, which in turn helped increase the yield of MA.

## 4. Conclusions

In conclusion, this report not only characterizes the high salt tolerance and optimal salinity for MA synthesis in the fragrance-producing yeast *T. ciferrii* WLW, but also reveals the molecular mechanisms underlying its salt tolerance and salt-driven fragrance production. *T. ciferrii* WLW exhibited a survival rate of 50.13% at 15% salinity, demonstrating that *T. ciferrii* WLW is an extremely halotolerant microorganism. The best performance of MA production by *T. ciferrii* WLW was achieved at 10% salinity, which emphasizes the favorable effect of an appropriate salt concentration environment on MA synthesis by *T. ciferrii* WLW. Statistical analyses based on generalized linear models showed that the time–salt concentration interaction effect significantly affected the ability of *T. ciferrii* WLW to synthesize MA. Transcriptome analysis revealed that membrane proteins, ion homeostasis, and transcription factors are important to salt tolerance in *T. ciferrii* WLW. Salt-stressed environments can affect cell survival and aroma production capacity by inhibiting the TCA cycle. Metabolic pathways such as the PPP, the shikimate pathway, and tryptophan biosynthesis formed important components of the molecular mechanism of salt-pressure-driven aroma production in *T. ciferrii* WLW, and key genes in these pathways acted together to help the flow of carbon towards MA synthesis, which resulted in an increase in MA production in *T. ciferrii* WLW. These findings facilitate future enhancements in the fermentation process of *T. ciferrii* WLW for MA production, particularly when integrated with response surface metabolomics and other advanced techniques. This research not only offers valuable insights for optimizing production methods and expanding the application of critical functional microorganisms in the manufacturing of traditional food products, but also lays a solid theoretical foundation for exploring the mechanisms behind flavor development in fermented foods and their broader applications.

## Data Availability

The original contributions presented in the study are included in the article/Supplementary Material, further inquiries can be directed to the corresponding authors.

## Supplementary Materials

The following supporting information can be downloaded at: https://www.mdpi.com/article/10.3390/foods13111593/s1, Figure S1: Difference in expression of genes related to iron and copper ion homeostasis; Figure S2: Differences in the timing of expression of key genes for MA synthesis; Figure S3: Bubble map of fold-change order of the main different genes included citrate cycle; Table S1. Effect testing of generalized linear model based on data from salt tolerance test; Table S2: Summary of trimming and read mapping results of the sequences generated from *T. ciferrii* WLW yeast with or without salt condition; Table S3: Transcription factors predicted by JASPAR database; Table S4: The main different genes included in citrate cycle.
