# Supplementary material for: Revealing the Mechanism of Aroma Production Driven by High Salt Stress in Trichomonascus ciferrii WLW
Source: Foods. 2024 May 21;13(11):1593. doi: 10.3390/foods13111593 (PMC11172348; doi:10.3390/foods13111593)
Supplement: Supplementary file 1 [file foods-13-01593-s001.zip › foods-2953501-supplementary.pdf]

Revealing the mechanism of aroma production driven by high salt stress in *Trichomonascus ciferrii* WLW

Fanying Xian [1,†], Lin Yang [1,2,†], Huaqing Ye [1], Jinlin Xu [1], Xiao-ping Yue [2,*] , Xiaolan Wang [1,*]

[1] School of health, and School of life science, Jiangxi Normal University, Nanchang 330022, China
[2] College of Chemistry and Chemical Engineering, Jiangxi Normal University, Nanchang 330022, China
* Correspondence: xiaoping@hbu.edu.cn (X. Yue); xlwang@jxnu.edu.cn (X.L. Wang)
† These authors contributed equally to this work

Supplementary Materials

A

gene id	D2	D4	D6
	log_2FC	log_2FC	log_2FC
gene5666	4.700	12.275	1.701
gene5665	2.007	3.468	-1.010
gene1407	1.973	14.412	0.000
gene0905	0.301	0.218	0.590
gene1584	-3.207	9.652	0.000
gene1275	1.617	0.722	-0.471
gene3477	-0.823	0.834	0.018
gene1002	-1.397	-1.609	-0.683
gene1834	0.045	-1.175	5.893
gene6391	-1.036	1.631	2.094
gene1343	-2.241	0.067	0.625
gene3809	-1.058	-0.384	-1.187
gene0469	5.116	3.537	1.083
gene0907	0.273	1.910	0.559
gene0605	0.187	0.027	0.117
gene5757	-1.027	-0.744	-0.940
gene1492	-0.298	-0.362	-0.572

B

gene id	D2	D4	D6
	log_2FC	log_2FC	log_2FC
gene5666	4.700	12.275	1.701
gene5665	2.007	3.468	-1.010
gene1407	1.973	14.412	0.000
gene0905	0.301	0.218	0.590
gene1584	-3.207	9.652	0.000
gene1275	1.617	0.722	-0.471
gene3477	-0.823	0.834	0.018
gene1002	-1.397	-1.609	-0.683
gene1834	0.045	-1.175	5.893
gene5654	-0.188	0.414	-0.226
gene3975	0.913	0.229	-0.081
gene6644	-0.704	0.294	-0.488
gene1246	-0.870	-0.099	-0.824
gene0557	0.565	-0.777	0.874
gene5110	-10.069	2.252	-0.287

log_2FC
> 1
0 ~ 1
-1~ 0
< -1

C

gene id	Name	log_2FC			Annotation
		D2	D4	D6	
gene0915	ATP2C	0.372	0.213	0.348	calcium ion transmembrane transport
gene0139		0.849	1.497	2.311	calcium channel activity
gene1393	chaA	0.459	0.768	0.414	cation transmembrane transporter activity
gene4528	STRN1_3_4	0.043	0.751	0.726	calmodulin binding

Figure S1. Difference in expression of genes related to iron and copper ion homeostasis. (A) Logarithmic difference multiple of iron ion homeostasis related genes; (B) Logarithmic difference multiple of copper ion homeostasis related genes; (C) Logarithmic difference multiple of calcium ion homeostasis related genes. (Note: Gene names highlighted in red indicate genes that are common to both iron and copper ion homeostasis.)

log_2FC
> 1
0 ~ 1
-1~ 0
< -1

gene id	Time / Name	D2	D4	D6
		log_2FC	log_2FC	log_2FC
gene2707	pgi	0.218	1.0384	0.8627
gene0464	zwf	0.8305	0.1812	0.0205
gene3566	tktA	1.1315	1.332	0.2469
gene1645	aroF	0.7627	0.1405	-0.41
gene2245	aroF	0.4394	0.4489	-0.561
gene6863	aroF	0.4254	0.173	-0.526
gene3855	ARO1	13.577	11.963	2.3904
gene2527	aroC	0.1818	0.2643	-0.542
gene6512	glnA	0.3287	-0.639	-2.274
gene1418	metK	1.4398	3.8718	-0.086
gene1200	trpE	0.2011	0.481	-0.093
gene4382	TRP3	-0.204	0.7284	-0.112
gene3948	trpD	-1.012	-0.576	-1.246
gene5406	hisA	-3.168	0.5153	-0.846
gene3045	INDO	-0.306	-0.619	-0.943
gene1267	kynU	-0.3	-0.987	0.1142
gene1630	kyn	-5.65	-1.084	0.5345
gene4352	BNA7	1.2737	0.9323	-1.184
gene6092		-2.064	-2.225	2.4075
gene0314		0.3224	0.3879	0.7083
gene3134		0.3301	-1.49	0.4728
gene3062	BNA3	0.3921	0.3417	-0.211

Figure S2. Differences in the timing of expression of key genes for MA synthesis.

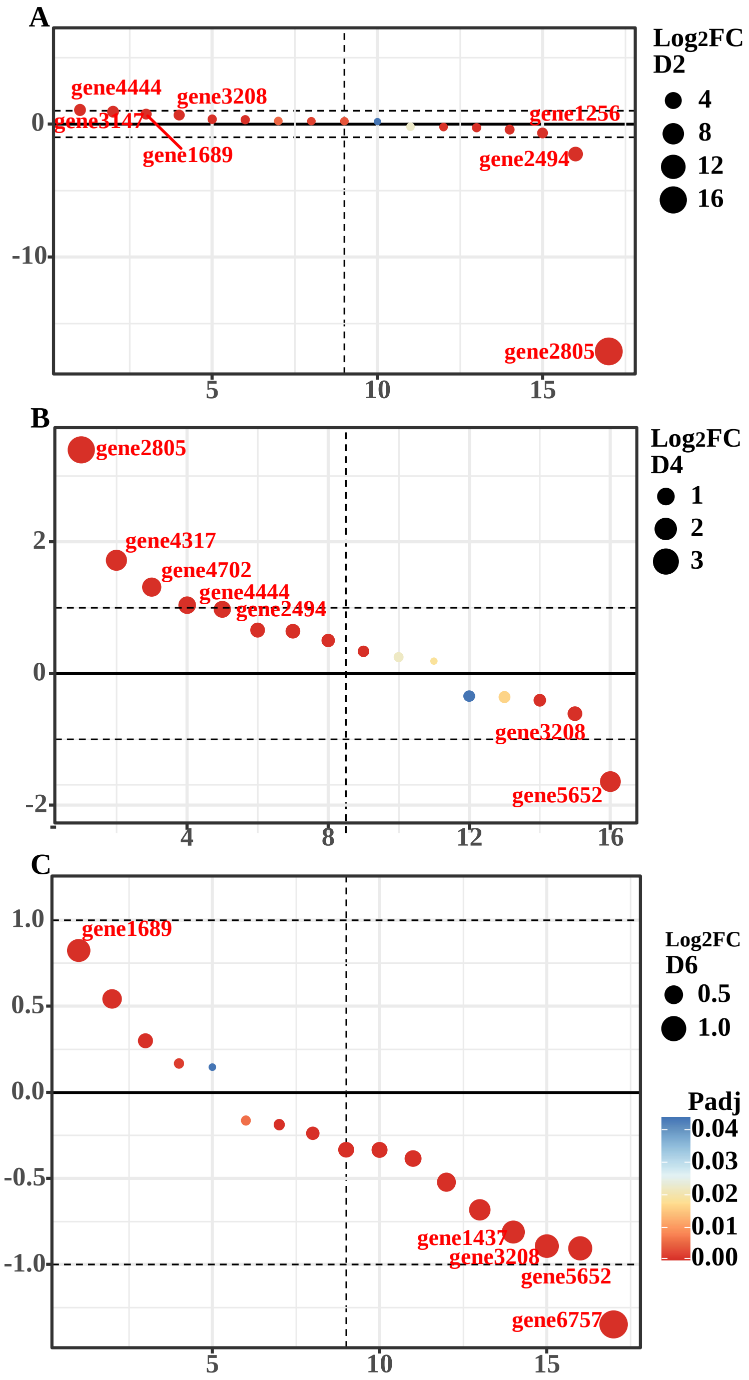

Figure S3. Bubble map of fold change order of the main different genes including in Citrate Cycle. A,B and C correspond to differential gene ordering on days 2, 4 and 6, respectively.

Table S1. Effect testing of generalized linear model based on the data from salt tolerance test

**Source**	**Type III**		
	**Wald Square**	**df**	**Sig.**
**Intercept**	48608.417	1	.000***
**Concentration of NaCl (C_{NaCl})**	15822.675	8	.000***
**Time**	349.975	2	.000***
**C_{NaCl} * Time**	590.451	16	.000***

***repsent p value <0.001.

Table S2. Summary of trimming and read mapping results of the sequences generated from WLW yeast with or without salt condition. (Salt concentration for salty conditions is 10%)

Sample	Raw reads	Clean reads	Q20(%)	Q30(%)	GC content (%)	Total mapped	Mapped rate (%)
A1	46794622	46474868	98.74	95.97	52.94	45566560	98.05
A2	46639378	46217078	98.6	95.64	52.98	45442061	98.32
A3	51469228	5101383 4	98.46	95.23	52.85	50063335	98.14
A4	47618156	47056408	97.98	94.36	52.6	45651413	97.01
A5	48551412	47872646	98.14	94.8	52.94	46553567	97.24
A6	45684012	45123020	97.9	94.16	52.58	43739850	96.93
A7	40687686	40162444	98.26	94.88	52.5	39319240	97.9
A8	50053874	49497154	98.32	95.04	52.18	48383567	97.75
A9	45121422	44673092	98.41	95.24	52	43664380	97.74
B1	46855532	46529340	98.59	95.6	52.75	45522893	97.84
B2	48131568	47681486	98.31	94.89	52.91	46660258	97.86
B3	43480310	43040408	98.38	95	52.91	42188297	98.02
B4	53090626	52654106	98.54	95.48	52.42	51654328	98.1
B5	46273784	45948046	98.49	95.3	52.28	44969456	97.87
B6	54124588	53695254	98.5	95.34	52.35	52652124	98.06
B7	56401044	55814542	98.36	95.04	51.71	54664291	97.94
B8	44964352	44490480	98.32	94.94	51.82	43586203	97.97
B9	45526268	45034920	98.27	94.81	51.75	44113954	97.95

Table S3. Transcription factors predicted by JASPAR database

Transcript id	Gene id	TF name	Motif id	Family
rna_gene6782	gene6782	ASH1	MA0276.1	C4-GATA-related
rna_gene5591	gene5591	DAL80	MA0289.1	C4-GATA-related
rna_gene0729	gene0729	GAT1	MA0300.1	C4-GATA-related
rna_gene4672	gene4672	GAT1	MA0300.1	C4-GATA-related
rna_gene5591	gene5591	GAT1	MA0300.1	C4-GATA-related
rna_gene4672	gene4672	GLN3	MA0307.1	C4-GATA-related
rna_gene5591	gene5591	GZF3	MA0309.1	C4-GATA-related
rna_gene2471	gene2471	HAP2	MA0313.1	Heteromeric CCAAT-binding
rna_gene2847	gene2847	MIG1	MA0337.1	Other factors with up to three adjacent zinc fingers
MSTRG.2080.1	gene4502	SPT15	MA0386.1	TBP-related factors
rna_gene4502	gene4502	SPT15	MA0386.1	TBP-related factors
MSTRG.2511.1	gene4906	ARG81	MA0272.1	None
MSTRG.2511.2	gene4906	ARG81	MA0272.1	None
rna_gene3785	gene3785	ASG1	MA0275.1	None
MSTRG.1693.1	gene0100	CAT8	MA0280.1	None
rna_gene0100	gene0100	CAT8	MA0280.1	None
rna_gene5326	gene5326	MCM1	MA0331.1	None
rna_gene5878	gene5878	NHP10	MA0344.1	None
rna_gene5056	gene5056	ROX1	MA0371.1	None
rna_gene4572	gene4572	YHP1	MA0426.1	None
rna_gene4572	gene4572	YOX1	MA0433.1	None

Table S4. The main different genes including in Citrate Cycle.

Gene id	Gene name	KO id	Annotation in KEGG database
gene2805	gltA	K01647	Citrate synthase
gene5652	IDH3	K00030	Isocitrate dehydrogenase
gene6757	SDH1	K00234	Flavoprotein subunit of succinate dehydrogenase (ubiquinone)
gene1437	SDH4	K00237	Succinate dehydrogenase complex, Oxidative phosphorylation
gene4766	sucA	K00164	2-oxoglutarate dehydrogenase
gene4317	fumC	K01679	Fumarase
gene4444	MDH2	K00026	Malate dehydrogenase
gene4702	lpd	K00382	Dihydrolipoic acid dehydrogenase, redox activity
gene1256	LSC1	K01899	Succinate-CoA ligase complex
gene3147	ACLY	K01648	ATP citrate (pro-S)-lyase
gene3208	IDH3	K00030	Isocitrate dehydrogenase
gene1689	acnA	K01681	Aconitate hydratase
gene2494	pckA	K01610	Phosphoenolpyruvate carboxykinase, Flow from the TCA cycle to gluconeogenesis
